# Differential expression of epithelial sodium channels in human RCC associated with the prognosis and tumor stage: Evidence from integrate analysis

**DOI:** 10.7150/jca.48970

**Published:** 2020-10-23

**Authors:** Shangfan Liao, Huaibin Huang, Fabiao Zhang, Dongming Lu, Shuchao Ye, Luoping Zheng, Yingming Sun, Yongyang Wu

**Affiliations:** 1 Department of Urology, Affiliated Sanming First Hospital, Fujian Medical University, Sanming365100, Fujian, PR. China.; 2 Department of Medical and Radiation Oncology, Affiliated Sanming First Hospital, Fujian Medical University, Sanming365100, Fujian, PR. China.

**Keywords:** Renal Cell Carcinoma, Epithelial Sodium Channel Proteins, Prognosis, Integrate Analysis

## Abstract

**Background:** Epithelial sodium channels are disputed in renal cell carcinoma, but its functions and effects on clinical outcomes are not well understood.

**Materials and Methods:** IHC and PT-PCR were used to detect ENaCα, β, γ, AVPR2, AQP2, and MR expression in the primary tumor and peritumoral tissues. GEPIA online tool was used to analyze the relationship between epithelial sodium channels and clinical-pathological characteristics. Tumor IMmune Estimation Resource online tool was used to investigate the immune profile relevant to epithelial sodium channels expression.

**Results:** Quantitative RT-PCR analysis revealed that ENaCα, β, γ, AQP2, and AVPR2 mRNA were decreased in the RCC, but there was no difference in MR mRNA expression between kidney and RCC (*p*=0.238). The IHC analyses showed that the intensely positive staining of ENaCα, β, γ, AVPR2, and AQP in the renal tubular and the attenuated in the RCCs. MR displayed moderate staining in both RCC and normal tissue. With the promotion of staging, the expression of AQP2, AVPR2, and MR reduced gradually and predicted a better prognosis. Although ENaCα, β, and γ were unable to associate with staging, we still observed a high expression of ENaCβ and γ displayed a poorer prognosis of RCC.

**Conclusions:** ENaCs shows an oncogene profile in RCC, drugs targeting epithelial sodium channel should be a possible therapeutic way to treat RCC. AVPR2 and MR exhibit an encouraging immunomodulatory function; patients with low expression of AVPR2 and MR may obtain more benefit from immunotherapy.

## Introduction

Renal cell carcinoma (RCC) represents the sixth most frequently diagnosed cancer in men and 10^th^ in women, accounting for 5% and 3% of all oncological diagnoses, respectively 1. As a common urologic tumor, RCC comprises approximately 2-3% of all human malignancies and exhibits the highly cancer-related mortality 2. Approximately 30% of patients are diagnosed with metastases, and an additional 20-40% of patients develop metastases after radical nephrectomy with curative intent 2. The outcome of patients with metastatic RCC (mRCC) is poor, with a median survival time of 10 to 21 months 2,3. The most optimal treatment for the patients is based on clinical prognostic factors. Usually, the Memorial Sloan-Kettering Cancer Center prognostic model is the most extensively used 4. Hyponatremia is commonly seen in patients with cancer and is associated with increased mortality and morbidity. Low sodium is associated with poor disease-free and overall survival after nephrectomy. It could act as a new independent prognostic and predictive factor in patients with mRCC 5,6.

Moreover, hyponatremia is independently associated with a worse outcome in mRCC patients treated with targeted agents 7, and on-treatment normalization of hyponatremia to normal sodium values associates with favorable outcomes 8. The impact of low serum sodium on the outcome of targeted therapy in mRCC has been reported 9. The mechanisms behind hyponatremia in RCC are not entirely understood. Ordinarily, the syndrome of inappropriate antidiuretic hormone (SIADH), which may be ectopically produced by tumor cells, was supposed to act as an important factor that increased water resorption in the distal tubule and lowered plasma osmolality^10^.On the other hand, the sodium channel in distal kidney nephron such as the epithelial sodium channel (ENaC), the mineralocorticoid receptor (MR), the aquaporin-2 (AQP2), and the arginine vasopressin receptor-2 (AVPR2) also act as central factors to sustain a hormonally controlled sodium and water reabsorption then to maintain the balance of osmolality in the kidney.

Interestingly, several studies have suggested that these epithelial sodium channel proteins played a role in tumor development and progression in recent 11,12. A significant role for the ENaC channels was found in tumor cells, particularly in proliferation, migration, and apoptosis. Up-regulation expression of ENaCα could promote cell migration in BeWo cells. ENaCγ could significantly decrease cell proliferation and increase cell apoptosis in the inner medulla, collecting duct cells 13,14. Simultaneously, the different expression of AVPR2 has been reported in many kinds of carcinoma (e.g., breast, pancreatic, colorectal and gastrointestinal cancers, and small-cell lung carcinoma.). AVPR2 activation increased cell proliferation of clear cell RCC cell lines, and AVPR2 abnormal expression was detected in ccRCC and associated with the tumor grade 15,16. AVPR2 antagonists or gene silencing reduced cell viability of 786-O and Caki-1, and also decreased RCC tumor growth in mouse xenograft models. It seems that these sodium channels and receptors not only involved in the maintenance of serum sodium but also engaged in the development of tumors.

It is still unclear the expression of these epithelial sodium channel proteins associated with the sodium metabolism in RCC. To detect the expression of these channels and receptors in RCC might provide a new perspective to understand the mechanisms of sodium metabolism inside the tumor and figure out how these sodium channels proteins affect the prognosis of renal cell carcinoma.

## Material and Methods

### Patients

127 patients diagnosed with RCC with stage I-III at the department of urology, affiliated Sanming First Hospital, Fujian medical university, between 2007 and 2010 were included in this study. We performed the nephron-sparing surgery or radical nephrectomy in strict accordance with the NCCN guidelines.

The Ethics Committee approved the study protocol of the Sanming First Hospital, and all samples were used after each patient provided full informed consent.

### Pathological diagnosis and sample preparation

Histological diagnosis was established according to the guidelines of the World Health Organization using hematoxylin-eosin (HE) staining. A pathologist divided the tissue block into malignant RCCs and normal peritumoral kidney tissues immediately after nephrectomy. One part was frozen at -80 °C directly for isolation of the mRNA. Whereas another part was fixed in OCT then store at -80 °C for immunohistochemistry (IHC) analysis.

### Haematoxylin-eosin staining

HE staining was performed for staging all the specimens. Two blinded independent pathologists observed the HE staining. When the pathologists disagreed about tumor stage and grade, they reviewed the slides in a non-blinded way.

### Quantitative real-time reverse transcription-polymerase chain reaction (RT-PCR)

Total RNA was isolated from normal kidney and RCC samples with an RNeasy mini-kit and RNase-free DNase set (Qiagen, Hilden, Germany), and approximately 0.5 μg extracted RNA was reverse-transcribed using First-strand cDNA Synthesis Kit for RT-PCR (Roche, Basel, Switzerland) with random primer.

Real-time RT-PCR assay was performed with a Smart Cycler System (Cepheid, Sunnyvale, California, USA) using SYBR Green-I as the fluorogenic dye (Molecular Probes, Eugene, Oregon, USA). 2 μL of the complementary DNA was added into a 25-μL reaction system of Ex-Taq RT-PCR version (Takara, Otsu, Japan) with 0.2 μM of each pair of gene-specific primers, and then subjected to 45 PCR cycles. The primer sequences were shown in Table 1. The thermal program was 5 seconds at 95 °C for denaturation, 20 seconds at 60 °C for annealing, elongation. The mRNA expression level was normalized as the ratio to that of β-actin in each sample. Amplified PCR products were electrophoresed on 2.5% agarose gel to verify.

### Immunohistochemistry staining

Frozen tissues embedded in OCT compound (TissueTek, Sakura, Tokyo, Japan) were sectioned into 8μm slices. Three to five sections of each sample were examined in each patient. After fixed in acetone, all slides were treated with 3% hydrogen peroxidase (Dako, S2001) for blocking endogenous peroxidase. Then, slides were incubated for 90 minutes at room temperature or overnight at 4 °C with primary antibodies. The primary antibody used were: anti-ENaCα (H-95):sc-21012, ENaCβ (H-190):sc-21013, ENaCγ (H-110):sc-21014, MR (N-17):sc-6860, AQP2 (C-17):sc-9882, AVPR2 (I-20):sc-18101 for 1:50 dilution (Santa Cruz Biotechnology Inc. CA, USA). For the negative control, the primary antibody was replaced by an antibody dilution buffer (Dako, S3022). After washing with TBS, slides were incubated with a streptavidin-HRP labeled second antibody for 60 minutes at room temperature and treated with substrate solution 3,3'-diaminobenzidine for the color reaction. After counterstaining with hematoxylin for 4 minutes, all these slides were viewed with a light microscope.

### Bioinformation analysis

A TCGA based database http://gepia.cancer-pku.cn was used to analyze the relationship between epithelial sodium channels and clinical-pathological characteristics. Tumor IMmune Estimation Resource online tool was used to investigate the immune profile relevant to epithelial sodium channels expression.

### Statistical analysis

All values in the text give the mean±SEM. Statistical analysis was performed using SPSS version 21.0. A Pair-sample *t*-test was applied to assess the statistical significance of the expression between the normal kidney and RCC tissue. Hochberg's one-way ANOVA was used to assess the difference between sodium channel protein mRNA expression and clinicopathological parameters. All *p* values were two-tailed, and those < 0.05 were considered statistically significant.

## Results

### ENaCs, AQP2, and AVPR2 expression are attenuated in RCC tissue

**Figure 1:**
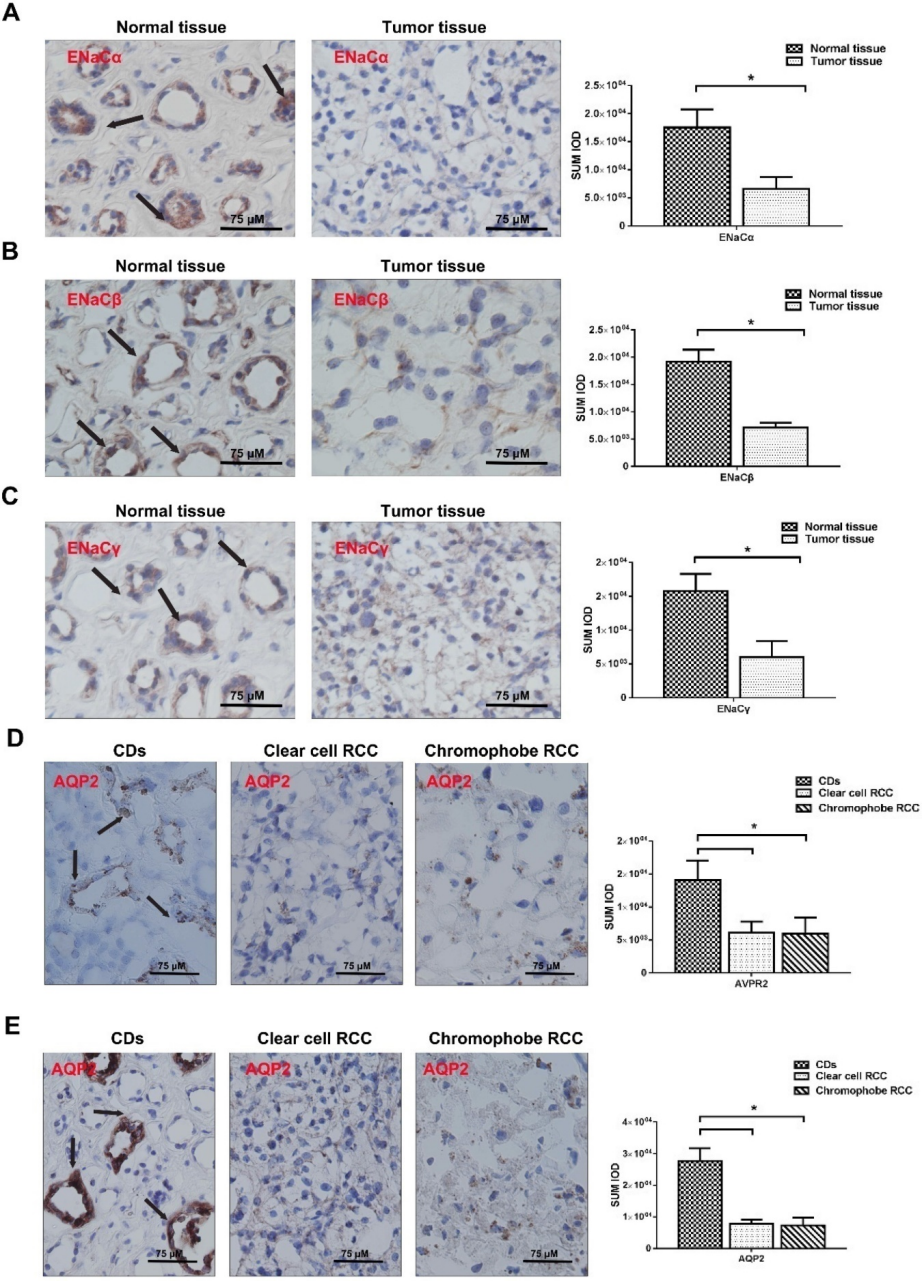
** ENaCs, AQP2, and AVPR2 expression is attenuated in RCC tissue. A.** The expression level of ENaCα protein was significantly lower in RCC tissues than those in the paired normal tissues and restricted to the tubular structures in normal peritumoral kidney tissue, especially CDs (black arrow),* p*<0.05. **B.** The expression level of ENaCβ protein was significantly lower in RCC tissues than those in the paired normal tissues and restricted to the tubular structures in normal peritumoral kidney tissue, especially CDs (black arrow),* p*<0.05. **C.** The expression level of ENaCγ protein was significantly lower in RCC tissues than those in the paired normal tissues and restricted to the tubular structures in normal peritumoral kidney tissue, especially CDs (black arrow),* p*<0.05. **D.** The expression level of AQP2 protein was significantly lower in RCC tissues than those in the paired normal tissues and restricted to the tubular structures in normal kidney tissues, especially CDs (black arrow), *p*<0.05. **E.** The expression level of AVPR2 protein was significantly lower in RCC tissues than those in the paired normal tissues and restricted to the tubular structures in normal peritumoral kidney tissue, especially CDs (black arrow),* p*<0.05.

The baseline and clinical-pathological characteristics were listed in Table 2. In general, the positive staining of ENaCα, β, γ, AVPR2, and AQP2 was restricted to the tubular structures in normal peritumoral kidney tissue, especially CDs. ENaCα, β, γ were strongly and diffusely presented in the cytoplasm of normal kidney CDs. Still, the relatively weak immunostaining was found in RCC (Fig. 1A, B & E). The differences were significant compared with the normal kidney. AQP2 was also strongly presented in the cytoplasm of normal kidney CDs. Still, only weakly positive staining was detected in RCCs (Fig. 1D). Similar to the AQP2, AVPR2 showed moderate and patching/focal immunostaining in the cytoplasm of the renal tubule, but displayed the weak/focal immunoreactivity in the RCCs (Fig. 1E).

### MR presents a similar expression in RCC tissue and normal tissue

**Figure 2:**
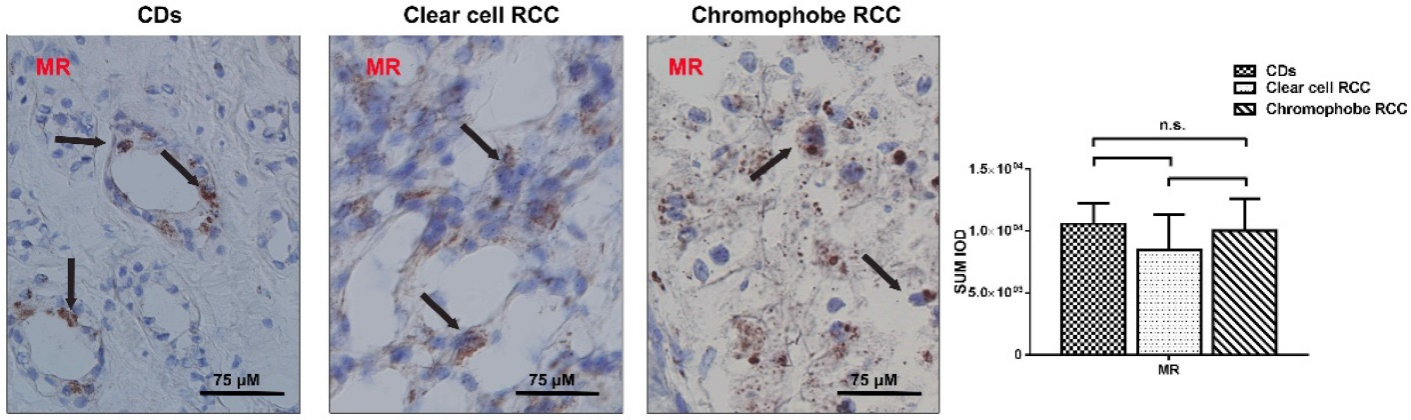
** MR presents a similar expression in RCC tissue and normal tissue.** The expression level of MR protein was similar in RCC tissues and those in the paired normal tissues and restricted to the tubular structures in normal peritumoral kidney tissue, especially CDs.

MR showed moderate and patching/focal immunostaining in the cytoplasm of the renal tubule and displayed a similar intensity and pattern of immunoreactivity in the RCC and normal kidney tissue (Fig. 2A).

### Epithelial sodium channels are unable to affect the clinical-pathological characteristics

**Figure 3:**
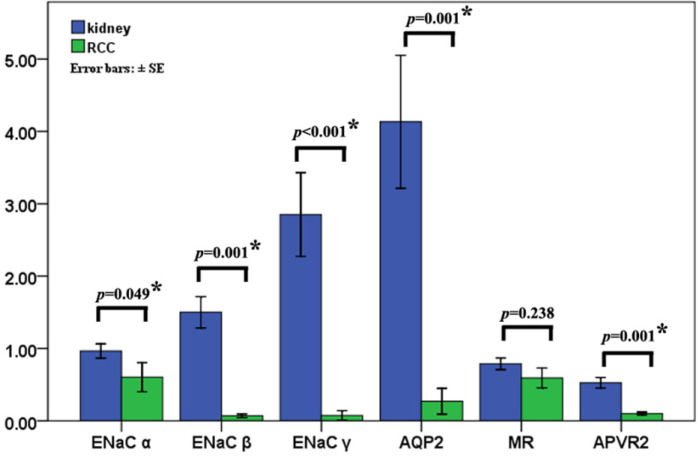
** Epithelial sodium channels are unable to affect the clinical-pathological characteristics.** The levels of T ENaCα, β, γ, AQP2, and AVPR2 mRNA were decreased 1.71, 23.52, 38.46, 5.18, and 15.28 times in the RCC compared with that in normal kidney using quantitative RT-PCR and the significant differences are detected. There was no difference in MR mRNA expression between kidney and RCC, *p<0.05.

Quantitative RT-PCR revealed that ENaCα, β, γ, AQP2, and AVPR2 mRNA were decreased 1.71, 23.52, 38.46, 5.18 and 15.28 times in the RCC (*p*≤0.05), respectively. There was no difference in MR mRNA expression between kidney and RCC (*p*=0.238) (Fig. 3).

We analyze the differences of quantified ENaCα, β, γ, AVPR2, AQP2, and MR mRNA expression among the age (≥59 years or <59 years), sex, pT-stage, Fuhrman grade, and histopathologic subtype. Unfortunately, we found epithelial sodium channels were unable to affect the clinical-pathological characteristics.

### ENaCα, β, γ, AQP2, AVPR2, and MR predicted various clinical outcomes of RCC

**Figure 4:**
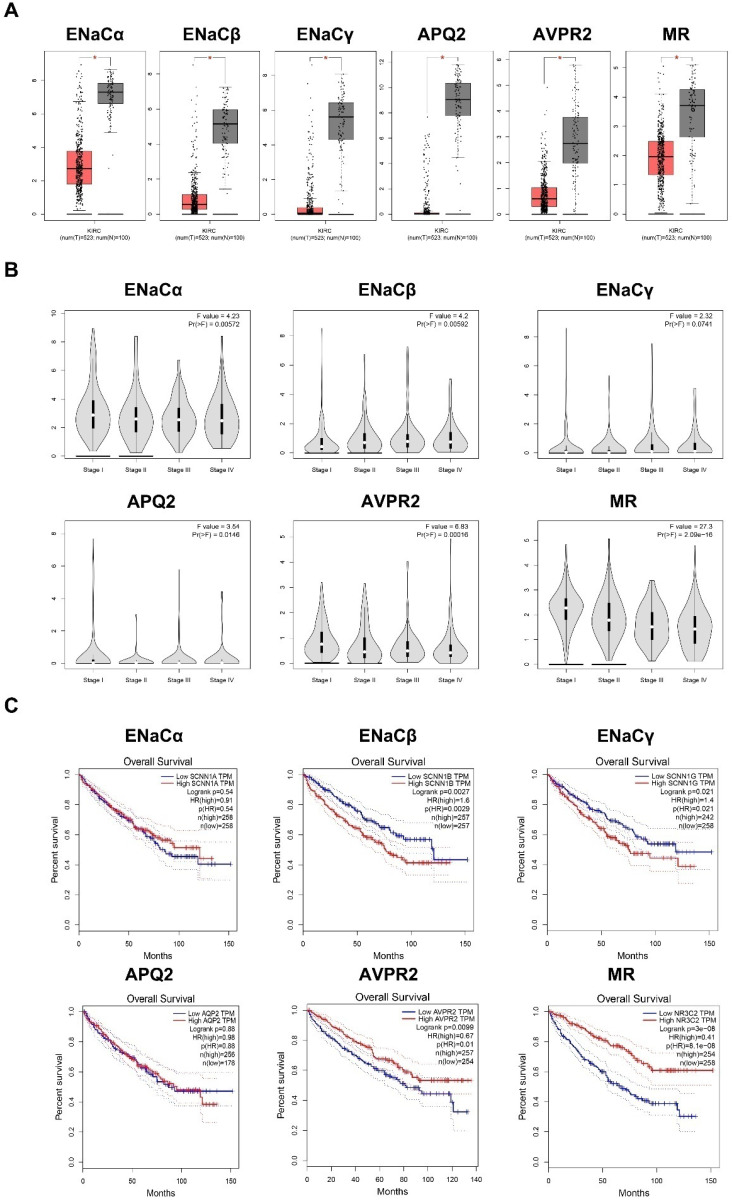
** ENaCα, β, γ, AQP2, AVPR2, and MR predicted various clinical outcomes of RCC. A.** The expression of ENaCα, β, γ, AQP2, AVPR2, and MR in the RCC tissues and normal peritumoral kidney tissue. **B.** ENaCα, β, γ, AQP2, AVPR2, and MR expression levels in RCC tissues subgrouped by staging. **C.** Kaplan-Meier plot with survival data stratified by ENaCα, β, γ, AQP2, AVPR2, and MR levels. All the data in this figure were captured from http://gepia.cancer-pku.cn/.

We searched the TCGA database to investigate further the potential effectiveness of ENaCα, β, γ, AQP2, AVPR2, and MR upon clinical-pathological characteristics in RCC. As shown in Figure 4A, the expression of ENaCα, β, γ, AQP2, AVPR2, and MR decreased in cancer tissue. Of note, with the promotion of staging, the expression of AQP2, AVPR2, and MR reduced gradually (Fig. 4B) and predicted a better prognosis (Fig. 4C). Although ENaCα, β, and γ were unable to associate with staging, we still observed a high expression of ENaCβ and γ displayed a poorer prognosis of RCC (Fig. 4C).

### ENaCα, β, γ, AQP2, AVPR2, and MR presented diverse immunological phenotypes of RCC

**Figure 5:**
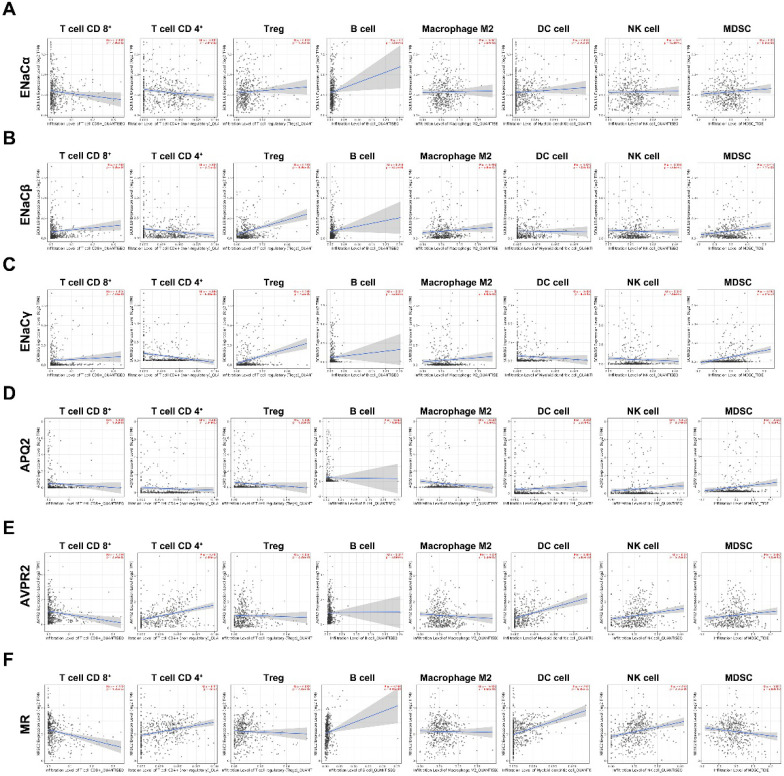
** ENaCα, β, γ, AQP2, AVPR2, and MR presented diverse immunological phenotypes of RCC. A.** The composition of different kinds of immune cells related to ENaCα in the TCGA KIRC database. **B.** The composition of different kinds of immune cells related to ENaCβ in the TCGA KIRC database. **C.** The composition of different kinds of immune cells related to ENaCγ in the TCGA KIRC database. **D.** The composition of different kinds of immune cells related to AQP2 in the TCGA KIRC database. **E.** The composition of different kinds of immune cells related to toAVPR2 in the TCGA KIRC database. **F.** The composition of different kinds of immune cells related to MR in the TCGA KIRC database.

To figure out immune cell infiltration profiles, we explored TIMER 2.0 online tool. A QUANTIseq algorithm was used to evaluate the composition of different immune cells in the TCGA KIRC database. As displayed in Figure 5A, ENaCα promoted Treg, B cell infiltrated; meanwhile, ENaCα attenuated T cell CD 8+, T cell CD 4+ infiltrated. Whereas ENaCβ advocated T cell CD 8+, Treg, B cell, Macrophage M2 infiltrated and debilitated T cell CD 4+ and DC cell infiltrated (Fig. 5B). For ENaCγ, we observed more Treg and MDSC infiltrated with less T cell CD 4+ infiltration (Fig. 5C). Instead, only the decrease of T cell CD 4+ infiltration is relevant to AQP2 (Fig. 5D). As shown in Figure 5E, AVPR2 expression was positively associated with T cell CD 4+, DC cell, and NK cell infiltration, while negatively associated with T cell CD 8+. Of note, we found MR had a similar immune cell infiltration profiles with AVPR2 (Fig. 5F).

## Discussion

In this article, we report the factors associated with sodium metabolism, including ENaCα, β, γ, AVPR2, AQP2, are significantly decreased in RCC. As the limitation of our sample amount, we cannot find the expression diversity of MR in RCC and normal kidney tissue; however, data from TCGA KIRC shows the expression of MR is attenuated in tumor tissue.

Although the sodium metabolism factors are attenuated in cancer tissue, we assume these factors reward different functions act related effects. Owing to their various affecting on staging and prognosis, the monism of sodium metabolism cannot explain the various phenotypes.

ENaCs, as sodium metabolism channels, should be the potential factors affecting tumor development and progression. As previously reported, the ENaCα deficiency could impair cell proliferation and migration in HepG2 and glioma cells 17. It seems to indicate that the ENaC could be involved in the process of carcinogenesis through affecting on Natrium influx. In this study, we find ENaC subunits deficiency would be more noticeable in those patients with large tumor size. The defections would probably cause the hyponatremia by decreasing the sodium reabsorption and increasing the sodium excretion. Furthermore, ENaCβ and γ are positively associated with more Treg and MDSC infiltration, which may be attributed to a poorer prognosis 18.

As previously reported, AVP could also increase the ENaC activity in the distal nephron. AVP could bind AVPR2 on the basolateral membrane of the collecting duct principal cells to stimulate the activity of adenylate cyclase, which subsequently increases intracellular cAMP levels and leads to the activation of PKA 19. In this study, we figure out AVPR2 expression is decreased in tumor tissue and relevant to a better prognosis. Of note, besides the activation of PKA, we find ACP may promote the T cells CD4+, DC, and NK cell infiltrated, which indicated better prognosis of RCC patients.

MR as homo-or heterodimers exist in nuclear and enhance ion and water transport to maintain blood pressure and lower potassium levels 20,21. However, many scholars reported MR as a tumor suppressor gene in various cancer types, including Warburg effect inhibition, VEGFA dysfunction, and EMT 22-24. Our findings partly confirm the above points. We demonstrate MR expression is positively associated with prognosis and early staging. Moreover, we reveal MR maybe module the tumor microenvironment *via* promoting the T cells CD4+, DC, and NK cell infiltrated. Meanwhile, MR also showed a potential function to scavenge Treg and MDSC in the tumor microenvironment. Altogether, MR attenuates RCC's progress in various ways.

Besides the typical role of osmotic transepithelial and transcellular sodium regulators, AQPs are also involved in carcinogenesis 25,26. They may facilitate angiogenesis, migration, tumor, and proliferation. However, AQP2 disruption was rarely reported. Mandinka reports AQP2 is independent of ovarian cancer 27. In contrast, Zhu et al. report AQP2 predicts a favorable prognosis in breast cancer 28. In this paper, we find AQP2 is decreased in tumor tissue; however, AQP2 cannot affect the staging and prognosis in RCC. Not to our surprise, bioinformation analysis display that AQP2 is failed to regulate immune cell constitution in the tumor microenvironment.

As the kidney has sufficient compensatory capacity, the hyponatremia is not very common in stage I-III patient 5, so we cannot provide the relationship among sodium channel expression, hyponatremia, and prognosis. But we convince sodium metabolism in RCC is disrupted, and should be a special issue for research. ENaCs shows an oncogene profile in RCC, drugs targeting epithelial sodium channel should be a possible therapeutic way to treat RCC. Apart from the classic function, AVPR2 and MR exhibit an encouraging immunomodulatory function. Patients with low expression of AVPR2 and MR may obtain more benefit from immunotherapy.

## Figures and Tables

**Table 1:** Primers used for RT-PCR

Gene	Primers	Lengths
**ENaC α**		
Forward	5'-ATG GAG TGG CCA AAG TCA AC-3'	200
Reverse	5'-GAG CAG CAT GAG GAACAT GA-3'	
**ENaC β**		
Forward	5'-TCC TGCAAT GAC ACC CAG TA-3'	199
Reverse	5'-TGG CTG CTG ATT CTT CAA TG-3'	
**ENaC γ**		
Forward	5'-GCT GAC CCT TCC ATT ACC AA-3'	200
Reverse	5'-TGC AGT CCC CTA TGC TAA CC-3'	
**AVPR2**		149
Forward	5'-CAC CCA TAC ACG TCT TCA TTG G-3'	
Reverse	5'-CAC CAT CTG CAG ATA CTT CAC G-3'	
**AQP2**		
Forward	5'-CCT CTA TTG CCC AGA TTG GA-3'	223
Reverse	5'-GGG GAA GCT TTG GAA ATA GC-3'	
**MR**		
Forward	5'-AAG GTA GAG TTC CCC GCA AT-3'	151
Reverse	5'-TGT GGA ACA ACA CAG GGA AA-3'	
**β-actin**		
Forward	5'-GGA CTT CGA GCA AGA GAT GG-3'	234
Reverse	5'-AGC ACT GTG TTG GCG TAC AG-3'	

**Table 2:** Summary of clinical and pathological features

Feature	Male	Female	Total
Patients	69	58	127
Age (years)	59.79±14.88	60.25±10.62	59.93±13.56
**Tumor Pathologic Stage**			
pT1a	23	21	44
pT1b	18	17	35
pT2	13	10	23
pT3	15	10	25
**Fuhrman Grade**			
Grade 1	26	22	48
Grade 2	26	20	46
Grade 3	17	15	32
Unclear	0	1	1
**Histopathology**			
Clear Cell RCC	49	42	91
Chromophobe RCC	7	3	10
Granular RCC	5	7	12
Papillary RCC	8	6	14

## Abbreviations

RCC: renal cell carcinoma
mRCC: metastatic RCC
AVP: arginine vasopressin
CD: collecting duct
ENaC: epithelial sodium channel
MR: mineralocorticoid receptor
AQP2: aquaporin-2
AVPR2: arginine vasopressin receptor-2
PKA: protein kinase A
HE: haematoxylin-eosin
RT-PCR: reverse transcription polymerase chain reaction
IHC: immunohistochemistry
SIADH: syndrome of inappropriate anti-diuretic hormone
